# Relationships between post operative pain management and short term functional mobility in total knee arthroplasty patients with a femoral nerve catheter: A preliminary study

**DOI:** 10.1186/1749-799X-6-7

**Published:** 2011-02-07

**Authors:** Catherine M Fetherston, Sarah Ward

**Affiliations:** 1 School of Nursing and Midwifery, Murdoch University, Education Drive, Mandurah 6210, Western Australia; 2 Peel Health Campus, Lakes Road, Mandurah 6210, Western Australia

## Abstract

**Background:**

Effective pain management following total knee arthroplasty (TKA) is fundamental in achieving positive rehabilitation outcomes. The purpose of our study was to investigate post operative pain management in relation to short term functional mobility in an intervention group receiving concomitant use of an IV narcotic PCA and a continuous infusion of local anaesthetic via a femoral nerve catheter (CFNC), compared to a group receiving narcotic PCA alone. This was a preliminary study conducted to establish an appropriate design for a larger investigative study.

**Methods:**

A prospective design was used to measure the effect of a CFNC on post operative pain management and functional mobility prior to hospital discharge. The amount of fentanyl used, pain and nausea scores, timed up and go (TUG) tests and active range of knee movement (AROM) were used to compare a CFNC and supplemental narcotic patient controlled analgesia (PCA) group (n = 27) with a PCA only group (n = 25).

**Results:**

The CFNC group used significantly less fentanyl than the PCA only group (p < .001) but there was no significant difference in TUG times between the two groups. There was however a significantly lower AROM reported for both extension (p < .04) and flexion (p < .006,) in the FNC group. Women had significantly slower TUG times (p < .005,) and there were moderate to strong positive correlations between post operative TUG times and the preoperative TUG time (r_s _= .505 p < .001), the time since oral analgesia (r_s _= .529 p < .014), and pain scores (r_s _= .328, p = .034)

**Conclusions:**

In this small preliminary study improved TUG performance at Day 4 post op was not influenced by the use of a CFNC but was positively correlated with male gender, preoperative performance, time elapsed since last oral analgesia and pain score. However AROM was decreased in the CFNC group suggesting further research on the relationship between CFNCs, local anaesthetic concentration and quadriceps strength should be incorporated in the follow up study's design.

## Background

Effective pain management following total knee arthroplasty (TKA) is generally believed to be fundamental in achieving positive rehabilitation outcomes. It has been shown to be important for early physiotherapy and increased mobility and has been identified as an influential factor in successful rehabilitation and reduced length of hospital stay [1,2]. However, a recent Danish study challenges this belief with their finding that pain has limited impact on functional recovery beyond the first post-operative day [3].

There are a range of methods that have been found to result in early effective postoperative pain control in patients undergoing TKA. These include intravenous (IV) patient controlled analgesia (PCA), intrathecal and epidural analgesia, lumbar plexus blockade, periarticular injection of local anaesthetics, single injection and continuous femoral nerve blockade, oral and intramuscular (IMI) narcotic and non narcotic analgesics [4]. Although IV narcotic PCA has been shown to be more effective than IMI and oral narcotic analgesia, and has the advantage of decreasing wait time for dose administration and dependency on nursing staff, it has been associated with significant adverse effects such as hypotension, confusion, pruritis, nausea and vomiting [5], all of which may potentially interfere with early postoperative mobility. Alternatively, the concomitant use of femoral nerve catheters (FNC) has been found to be associated with less PCA use, lower pain scores, and a shortened length of hospital stay [6-9] and as a result is becoming more appealing as a form of post operative analgesia. This popularity may not be justified in regard to continuous femoral nerve catheters (CFNC) as a review by the PROSPECT group (2008) concluded that although a femoral nerve block (FNB) was recommended based on evidence of reduced pain scores and supplemental analgesia, benefits of a continuous infusion compared to a single injection FNB may not be sufficient to justify the placement of catheters on a routine basis [10]. Further to this, a recent study has also found that extending an overnight CFNC to four days did not result in any increase in health related quality of life at either seven days or 12 months despite an increase in passive knee flexion during the infusion period [9,11]. Additionally, evidence in relation to the possible effects of continuous femoral nerve infusion on short term functional recovery is mixed. Two studies, one a retrospective pilot study [7] and the other a prospective randomised study [12] reported improved outcomes in the CFNC group in regard to increased flexion [7,12] decreased mobility assistance requirements [7] and decreased length of hospital stay [7]. Whereas a further two randomised controlled studies [13,14] reported no difference in either maximal knee flexion on post operative day (POD) 1 and 2 [13] or at discharge [14], or in the 2 min walk test conducted on PODs 1 to 3 [13]. There was also no difference in either time to first ambulation or length of hospital stay [14].

The purpose of our study was to investigate post operative pain management in relation to short term functional mobility in an intervention group receiving concomitant use of an IV narcotic PCA and a continuous infusion of local anaesthetic via a FNC, compared to a group receiving narcotic PCA alone. This was a preliminary study conducted to establish an appropriate design for a larger investigative study.

The research questions for this preliminary study were:

1. Is postoperative pain management improved in TKA patients who have a CFNC in addition to an intravenous narcotic PCA compared to patients whose pain is managed with narcotic PCA alone?

2 Is there a difference in expected and experienced levels of pain between the two comparison groups?

3. Is functional mobility at discharge improved in patients whose post operative analgesia is managed with concomitant use of an intravenous narcotic PCA and CFNC compared to those patients managed with narcotic PCA alone?

Outcome measures used in this preliminary study included patient's perceived pain, narcotic usage, and short term functional mobility. Functional mobility outcomes were measured using active range of knee movement (AROM) and a timed up and go test (TUG), prior to discharge.

## Methods

### Design

A quasi-experimental design was used where patients at a regional acute care hospital in Western Australia, who were undergoing a primary TKA, were followed prospectively to the fourth day (Day 4) after their surgery. University and hospital ethics committees' approvals were obtained prior to sequential sampling of those patients booked for a primary Press-fit TKA under the one orthopaedic surgeon. Recruitment of participants was undertaken between June and November 2008 and 64 patients were invited to take part in the study pre-operatively, either at the pre-admission clinic or on the surgical ward at the time of admission. Fifty seven patients consented to participate however five withdrew from the study post-operatively. This resulted in a final sample number of 52, of which 51.9% percent (n = 27) were managed by anaesthetist 1, who used a continuous infusion of ropivacaine 0.2% via femoral nerve catheter in conjunction with supplemental IV fentanyl PCA. The remainder of the sample which constituted the PCA only group was managed by anaesthetist 2 who used only, either fentanyl PCA for 40.4% (n = 21) or morphine PCA for 7.7% (n = 4) of the group. As Anaesthetists 1 and 2 operated pre-admission clinics on alternate weeks, allocation of study participants to either the CFNC and supplemental PCA group or the PCA only group was not randomised, but occurred sequentially according to the order and timing of when booking documentation was received at the clinic.

In Anaesthetist 1's group a FNC was inserted post induction of anaesthesia, and prior to incision, via the guidance of a nerve stimulator technique. A loading dose of 20 ml 0.75% ropivacaine and 20 ml 2% lignocaine was administered in the recovery room prior to commencement of continuous infusion of 0.2% ropivacaine. Intravenous PCA for both groups was inserted in theatre and commenced either in recovery or on return to the ward depending on patient demand for analgesia.

A physiotherapist assisted all post operative patients to stand out of bed on Day 1, the day following surgery, and mobilisation rehabilitation began on Day 2 and was supervised twice a day. Continuous Passive Movement (CPM) was commenced on the evening of Day 1 and was then supervised twice a day until discharge. Angle of flexion for CPM was aimed at achieving 30^0 ^on Day 1, 45^0 ^on Day 2, 60^0 ^on Day 3 and 90^0 ^on Day 4.

### Data collection/Instruments

#### Outcome measures for pain management

Post operative perceived pain was recorded during routine post operative observations by nursing staff, who asked participants to self report their pain using a verbal rating scale (VRS) of 0-10 where 0 = no pain and 10 = the worst pain imagined. Additionally a short questionnaire containing Likert scale questions designed to describe the expectations and experience of the participant's post operative pain was administered to participants on Day 4 post operatively.

#### Outcome measures for functional mobility

TUG tests and active range of knee movements (AROM) were both measured preoperatively (TUG1), at the time of recruitment, and then again post operatively on Day 4 (TUG2). The TUG2 s were measured either on the ward or in the physiotherapy department prior to the morning rehabilitation session. The TUG test, first described by Podsiadlo and Richardson [15] is a simple indicator of an older adult's functional mobility. It involves timing how long it takes the participant to stand up from a chair (seat height approximately 46 cm and arm height approximately 65 cm from the ground), walk 3 metres, turn and walk back to the chair and sit down. Results for TUG tests are categorised as worse than average if they exceed 9.0 secs for 60-69 years, 10.2 secs for 70-79 years and 12.7 secs for 80 to 99 years [16]. TUG tests were undertaken pre and postoperatively by the researchers or research assistant.

Preoperatively participants used a gait aid if this was how they normally mobilised. Crutches were used by 7.7% (n = 4) and a Zimmer frame by 3.8% (n = 2). Postoperatively elbow crutches were encouraged as the aid of choice although 25% (n = 13) used a Zimmer frame as they had not yet developed confidence using crutches. Reliability and validity have been established previously [15,17,18] and the inter-rater reliability for TUG test timing between researchers in this study was measured using pairwise correlation (r = 1.0, p < .001, n = 10). Range of knee movement was measured by one of the three treating physiotherapists using a universal goniometer. Intra and inter-rater reliability of the goniometer has been established previously using Pearson's intra-class correlation (ICC _(1.1) _= 0.99, 0.90) [19].

#### Demographic variables

Demographic data and data on daily CPM, pain and nausea scores and type and doses of analgesia, local anaesthetic and anti-emetics received were collected from the patient's medical record, in addition to the number of PCA attempts by the patient versus the number of PCA delivered doses. As a wide range of anti-emetics were prescribed by the two anaesthetists managing each group, nausea score was used as an outcome measure of nausea and vomiting in preference to amount of antiemetic used. Postoperative vomiting and nausea was classified as either: none (0), mild - intermittent nausea (1), moderate - dry retching (2) or, severe - vomiting (3).

### Data Analysis

Statistical analysis was conducted using Statistical Package for the Social Sciences (SPSS 17^®^) for Windows (2008) [20]. Descriptive statistics were reported as mean and standard deviation (SD) or median and interquartile range (IQR) according to normality. As data were mixed in regard to normality a Mann Whitney *U *test was used to compare data from the FNC and PCA only groups. Effects size were calculated as recommended by Clark-Carter [21] and classified by Cohen's [22] conventions where an r = .1 can be considered a small effect size, r = .3 a medium effect and r = .5 a large effect. Correlational analyses were conducted using Spearman's Rho (r_s_) for ordinal and non-normal scale data . *P *values less than 0.5 were considered significant for all data.

## Results

### Demographic data

The sample consisted of 44.2% (n = 23) male and 55.8% (n = 29) female patients with more females than males in the CFNC group (n = 17 and 10 respectively), than in the PCA only group (n = 12 and 13 respectively). Age was similar for both groups within the sample with a mean of 70 ± 8.6 years for the CFNC group and 70 ± 7.3 years for the PCA only group, as was body mass index, (29 ± 5.5 for the CFNC group and 30 ± 3.9 for the PCA only group). A right TKA was performed for 61.5% (n = 32) of participants, and a left TKA for 38.5% (n = 20) with similar representation in the CFNC group (left = 10, right = 17) and the PCA only group (left = 10, right = 15). Mean degrees of flexion reached using CPM for each post operative day was not significantly different for either group (Table 1) and there was no significant difference in average length of stay which was 6.0 ± 1.46 nights for the CFNC group and 6.0 ± 0.68 nights for the PCA only group. Patients in the CFNC group received a mean total dose of 354 ± 71 mL of ropivacaine 0.2% over a mean duration of 36 ± 5.8 hours at an average hourly dose of 10 ± 2.4 mL.

**Table 1:** Comparison of post operative flexion (mean ± SD) attained using CPM for the FNC and PCA only groups

Day post op	FNC and PCA group	PCA only
Day 1 Flexion (degrees)	30.3 ± 7.5	29.4 ± 7.7
Day 2 Flexion (degrees)	43.8 ± 10.4	46.3 ± 7.1
Day 3 Flexion (degrees)	66.2 ± 6.9	67.1 ± 7.8
Day 4 Flexion (degrees)	83.3 ± 7.3	80.2 ± 6.8

### Narcotic analgesia and pain scores

There was no significant difference in the duration of PCA in the CFNC and PCA only groups (Table 2) and the amount of intravenous fentanyl used by both groups was compared. There was significantly more total and daily fentanyl used in the PCA only group compared to the CFNC group. This was also true for fentanyl used/BMI. There were also significantly more dose increases required on the day of surgery in the PCA only group (Table 2).

**Table 2:** Comparison of fentanyl PCA data for patients with and without a FNC

	FNC group (n = 27)	PCA only group (n = 21)	Statistical significance
PCA duration (hours)	*37.7 ± 2.8	39.6 ± 4.4	NS
Total fentanyl used (μg) Day 0	450 (220, 73)	820 (609, 1165)	p < .001 Effect size medium, r = .48
Total fentanyl used (μg) Day 1	1120 (460, 1560)	1388 (1080, 2329)	p < .067 Effect size small, r = .23
Total fentanyl used (μg) Day 2	180 (120, 295)	237 (188, 496)	p < .03 Effect size medium, r = .31
Total fentanyl (μg) used	1860 (800, 2556)	2820 (2356, 4238)	p < .001 Effect size large, r = .52
Fentanyl used (μg)/hour	51.8 (22.8, 66.8)	89 (61, 104)	p < .001 Effect size large, r = .52
Fentanyl dose (μg)/BMI	53.6 (31.6, 93.8)	101 (71,129)	p < .002 Effect size medium, r = .45
Mean number of dose increases required on Day 0	0 (0, 1)	1 (0,1)	p < .016 Effect size medium, r = .35
% successful PCA attempts (Day 0)	75 (49, 89)	75.3 (58.3, 91.8)	NS
% successful PCA attempts (Day 1)	81.3 (62.8, 92.5)	83.6 (63.2, 89.4)	NS

*Mean ± SD, or median (IQR: 25,75), reported according to normality

The percentage of pain scores above 3, nausea scores above 0 and the highest reported pain scores on Day 2 were higher in the PCA only group although the difference was not significant. However the highest reported pain scores on both Day 0 (day of surgery) and Day 1 (day after surgery) were significantly higher (Table 3).

**Table 3:** Comparison of post operative pain and nausea and vomiting scores

Post operative pain and nausea scores	FNC group (n = 27)	PCA only group (n = 21)	Statistical significance
Average pain score (VRS)	3.8 (2.6, 4.6)	3.4 (2.7, 4.8)	NS
% pain scores above 3	45.3 ± 24.3*	48.5 ± 25.9	NS
Day 0 highest reported pain score	7.2 ± 1.7	8.36 ± 1.7	p < .017 Effect size medium, r = .35
Day 1 highest reported pain score	6 (6,7)	8 (6,9)	p < .034 Effect size medium, r = .31
Day 2 highest reported pain score	3.6 ± 2.1	4.3 ± 2.8	NS
% PONV scores above 0 **	3.3 (3,12.5)	6.4 (0,14)	NS

*Mean ± SD, or median (IQR: 25,75), reported according to normality

**Postoperative nausea and vomiting (PONV) was classified as either: none (0), mild - intermittent nausea (1), moderate - dry retching (2) or, severe - vomiting (3).

### Pain expectations

The pain experienced by patients was either more, or a lot more, than expected in 48.1% (n = 25) of the sample however there was no significant difference (*U *= 266, z = -.219, p < .826) in the degree of perceived pain in relation to expectations between the CFNC and the PCA only groups.

### Functional mobility

There was no significant difference in the type of gait aid used for either the CFNC or the PCA only group either pre- or postoperatively or in the preoperative TUG scores which were 12.5 ± 4.4 secs for the FNC group and 12.3 ± 3.7 for the PCA only group. Although the CFNC group had quicker postoperative TUG times the difference did not reach significance for this sample (Table 4) however there was significantly lower range of movement reported for both extension and flexion for the FNC group. There was no significant gender influence on AROM for either group (Table 5).

**Table 4:** Comparison of Day 4 Range of Movement, TUG and pain scores prior to TUG, for patients with and without a FNC

Functional mobility, Day 4 post operatively	FNC (n = 27)	PCA only (n = 25)	Statistical significance
AROM Flexion Day 4 (degrees)	70.8 ± 17.3*	82.6 ± 15.8	p < .006 Effect size medium, r = .38
AROM Extension Day 4 (degrees)	-11.9 ± 7.0	- 8.38 ± 4.4	p < .040 Effect size small, r = .29
TUG test post op (secs)	45.23 (31, 67)	58.0 (39.5, 71)	NS (outliers removed)
Pain score at TUG test	4.69 ± 2.3	3.67 ± 2.5	NS
Time elapsed since last analgesia at TUG test (hours)	*3.6 ± 1.6	2.0 ± 1.1	p < .003 Effect size medium r = .42

*Mean ± SD, or median (IQR: 25,75), reported according to normality

**Table 5:** Median (IQR) pre and post operative TUG test results (secs) and mean (SD) AROM (degrees) according to gender

	FNC group		PCA only group	
	**Male**	**Female**	**Male**	**Female**

Pre-op TUG (secs)	9.9 (9, 11.2)	13 (10, 14.1)	10.3 (9.4, 15.6)	11.5 (9.6,16.1)
Post-op TUG 2 (secs)	34 (30.9, 47.3)	60.7 (44.6, 97.5)	46.2 (34.5, 64.8)	68.5 (50, 79.6)
Post-op Flexion	*72.4 ± 11.9	71.5 ± 20.3	83.1 ± 15.3	82.1 ± 16.9
Post-op Extension	-11.6 ± 7.9	-12.2 ± 6.5	-8.5 ± 4.2	-8.27 ± 4.7

*Mean ± SD, or median (IQR: 25,75), reported according to normality

In the sample as a whole, men had quicker preoperative (*U *= 212.5, z = -1.91, p < .056, medium effect size, r = .30) and postoperative TUG times (*U *= 123, z = -2.78, p < .005, medium effect size, r = .42) than the women (Table 5). Men in the FNC group were also significantly quicker (*U *= 28, z = -2.04, p < .042, small effect size, r = .28) and approached significance for the PCA only group (*U *= 31, z = -1.91, p < .056, small effect size, r = .26).

There were moderate to strong positive correlations between postoperative TUG (TUG2) scores and preoperative TUG (TUG1) scores (r_s _= .505 p < .001) and the pain scores measured prior to TUG2 (r_s _= .328, p = .034), i.e. the faster the pre-op TUG time the better the patients performed post operatively; and the higher their pain score just prior to undertaking TUG2 the slower they performed. Additionally, the time lag since last pain medication prior to performing TUG2 was significantly higher for the FNC group (Table 4). This is reflected in a strong positive correlation (r_s _= .529 p < .014) in the FNC group between time since last medication and TUG2 scores and a strong negative correlation (r_s _= -.505 p < .023) for the no FNC group (i.e. the lower the time lag between analgesia and performing TUG2 the better they did). However there was no such correlation between range of movement and time since last pain medication for either group (FNC group, Extension: r_s _= -.01 p = .969, Flexion: r_s _= .098, p = .69; the no FNC group, Flexion: r_s_= -.089 p = .745, Extension: r_s = _-.084, p = .749).

## Discussion

Femoral nerve catheters are now used more frequently following TKA due to their improved efficacy in relation to lower pain scores, and an associated decrease in narcotic use post operatively. The benefits afforded by improved pain management related to the use of CFNCs, may also extend to benefits in short term functional recovery [7-9,23] and this study has sought to contribute further knowledge in this area by investigating the relationship between the use of a CFNC and patient recovery in terms of their pain management, and functional mobility prior to discharge from hospital. Both the CFNC and the PCA only groups compared in this study had similar characteristics in regard to age, body mass index, operation site, length of hospital stay and whether a gait aid was used pre-operatively. There were also no significant differences in either the type of gait aid used postoperatively or in flexion attained when performing CPM exercises, whilst in hospital. Intravenous narcotic PCA was available to patients in both groups for a similar duration and both groups appeared to have a similar understanding of how to use PCA, as evidenced by comparable percentages of successful PCA dose attempts delivered. However there was a higher representation of women in the CFNC group than in the PCA only group.

This study's results have supported previous findings that patients with a CFNC use less supplemental narcotic analgesia during the postoperative period [6,24,25]. Having a CFNC in situ had a significantly large effect on the total fentanyl used with lower doses used, not only on the day of operation, but also on the first and second days postoperatively. The amount of fentanyl used also remained significantly lower when BMI was taken into account (Table 2). In general, patients in both groups appeared to manage their pain appropriately with an average pain score below 4 for both groups, however the highest pain score reported by patients was significantly higher (medium effect size) in the PCA only group on both the day of, and the day after, the operation. Although nausea scores were higher in the PCA only group, the difference did not reach significance in this sample and this is comparable with findings from previous studies [6,24,26].

Short term functional mobility was measured using a TUG test and AROM on Day 4 following surgery, which was the day prior to assessment for hospital discharge on the clinical pathway. In a study that examined physical performance measures after TKA [18] the TUG test was found to be useful in the early recovery period between one and nine to ten weeks postoperatively, by which time the test had reached a ceiling effect where most patients had met the 10 second criteria for being functionally independent [15]. Preoperative TUG results in our preliminary study indicate a degree of disability prior to surgery with the sample averaging a time of 12.4 seconds. This was greater for women, who had slower TUG times and supports previous research that found gender differences exist, with women reporting greater disability at the time of arthroplasty and lower self-reported function [18,27,28]. This may be explained by a study that found that women undergo arthroplasty at a more advanced disease state than men and also, as a result, had reduced muscle activation and increased atrophy preoperatively [27]. This level of disability was also reflected for women in our study's post operative TUG scores with women again having significantly slower times than men. It should also be noted that the researchers subjectively observed the men to be very competitive when completing their TUG tests, often asking the researchers how they compared to others and then "swapping results" on return to their shared rooms. Despite the gender influence, and the higher proportion of women in the FNC group, median TUG times were quicker for the FNC group (45 secs) than the PCA only group (58 secs), although the difference was not significant for this sample.

In comparison, the AROM was significantly lower in the CFNC group than in the PCA only group. Support for this finding is mixed. These results are not supported by previous findings from studies by Kadic et al. [12] and DeRuyter et al. [7] who found increased flexion on Days 3 to 6, and at Day 1 and discharge, respectively, in patients with a CFNC in situ for 48 hours. However a study by Carli et al. [13] using only a slightly lower dose regimen of 8 ml/h, compared to the10 ml/h of 0.2% ropivacaine in this study, found no significant difference in knee flexion on Days 1 and 2. The results in our study were not explained by the significant difference in the time analgesia was administered prior to testing, or by gender differences in each group. Most likely they can be explained by studies that found that quadriceps strength is the strongest predictor of functional performance [29,30]. This is a matter for concern in regard to short term functional mobility considering the recent published caution that femoral nerve blockade may result in prolonged quadriceps weakness and an increased risk of falls [31]. The variance in our results compared to the short term improvements seen in the study by Kadic et al. [12] may be related to a reduced dose of ropivacaine received by participants in their study. They described a dose of between 5 and 10 ml/h of 0.2% ropivacaine for the first 48 h (as opposed to an average dose of 10 ml/h in this study) although there were no details of the mean hourly or total dose received by participants making comparisons between the studies difficult. However recent data on the minimum local anaesthetic concentration (MLAC) showed that the minimum concentration at which patients did not require rescue analgesia using levobupivicaine was 0.024% for the femoral nerve and 0.014% for the sciatic nerve [32]. Even with this ultra-low concentration, which equates to a reduction in the commercial preparation's concentration of four to six fold, there was mild motor block manifested by an inability to dorsiflex, which prevented early mobilisation. This may indicate that the doses of ropivicane used in this study are related to the significantly lower AROM observed. However this may not be significant in terms of long term recovery as Kadic et al. also observed that short term improvements in knee flexion did not correlate with increased knee flexion and improved functional outcomes at three months.

The preoperative decline of quadriceps' strength and function has been shown to impact negatively on functional recovery [33,34] and may also explain our findings of a strong positive correlation between slower preoperative TUG times and poorer postoperative TUG performance. However the correlations between slower TUG times and an increased time between analgesia and exercise, and a higher pain score, also reinforces the importance of timely effective analgesia prior to undertaking exercise in the post operative period prior to discharge. Our findings suggest that oral analgesia prior to exercise, once the femoral nerve catheter has been removed, is influential in improved functional mobility although this is at odds with recent findings from Denmark that pain has limited impact on functional recovery past the first postoperative day [3].

Positive impact on functional recovery may also be achieved through earlier surgical intervention to prevent severe disability prior to surgery [34-36]. However, TKA is often delayed by either wait lists or the requirement to decrease the need for future revision arthroplasty. In these cases improved preoperative function through the use of physical therapies has been shown to be effective in improving postoperative function [37].

### Study limitations and considerations for the follow-up study

As a preliminary study this design was limited by the lack of probability sampling and small sample number. Subsequently there was a difference in gender representation with the increased proportion of females in the CFNC group being more representative of the general population, than in the PCA only group. This emphasises the importance of conducting a randomised design with an a priori power analysis in the follow-up study. Despite the absence of these design features in this preliminary study, the comparison groups were similar in their characteristics and the study was conducted prospectively with differences between groups identified as having medium to large effect sizes, so enabling important information for the conduct of the follow up study.

## Conclusions

This study found that a CFNC was associated with important short term pain management benefits whilst insitu, in terms of significantly less supplemental narcotic use, and a lower number of narcotic PCA dose increases. Although the average pain score was not significantly different for the CFNC and PCA only groups the highest reported pain score was higher in the PCA only group on both the day of, and the day after the operation. The similar average pain scores reported in both groups is probably indicative that patients managed their pain appropriately with the assistance of rescue PCA, and that the CFNC patients required significantly less supplemental IV narcotic to achieve this. However the benefits observed for the CFNC whilst in situ, did not appear to extend to improved functional mobility after its removal. Three factors associated with reduced functional mobility on Day 4 post operatively were identified in this preliminary study. They were: gender, with women demonstrating slower TUG times; an increased time since oral analgesia was administered prior to mobilisation, which also accompanied a higher pain score; and a higher level of preoperative disability. This reinforces previous research that suggests there are several important factors that influence the short term post operative functional recovery after TKA. These include timely and effective analgesia prior to postoperative exercise and either timely surgery before marked muscle deactivation and atrophy occurs, or alternatively improving function prior to surgery using physical therapy. Of concern was the finding that AROM was significantly decreased in the CFNC group. This indicates that CFNC may be a variable of influence in the short term post-operative functional mobility of patients, especially in regard to concentrations of local anaesthetics used and its effects on quadriceps strength, and is therefore an important consideration in the follow up research.

## Abbreviations

TKA: total knee arthroplasty; CFNF: continuous femoral nerve catheter; PCA: patient controlled analgesia; TUG test: timed up and go test; AROM: active range of motion; CPM: continuous passive movement; MLAC: minimum local anaesthetic concentration

## Competing interests

The authors declare that they have no competing interests.

## Authors' contributions

CF conceived and designed the study, assisted with the data collection, performed the statistical analysis and prepared the manuscript

SW participated in the design of the study, data collection and manuscript preparation
